# Effort-reward imbalance among medical students and physicians

**DOI:** 10.1186/1472-6963-14-S2-P143

**Published:** 2014-07-07

**Authors:** Lucia Jerg-Bretzke, Manuel Fenkl, Harald C Traue, Kerstin Limbrecht-Ecklundt

**Affiliations:** 1 University of Ulm, Medical Psychology, Frauensteige 6, 89075 Ulm, Germany; 2 University of Hamburg, Outpatient Clinic for Behavioral Therapy, Von-Melle-Park 5, 20146 Hamburg, Germany

## Background

Effort-reward imbalance (ERI) is recognized as a risk factor for work stress and burnout. In the process of becoming a doctor, students of medicine face different challenges compared to practicing medics. In this study we focus on how the different challenges of study life and work life of medics have a bearing on the effort-reward imbalance.

## Materials and methods

The questionnaire used for the practicing medics was the effort-reward-imbalance (ERI) model of Siegrist *et al.* The questionnaire used for the students survey was the effort-reward-imbalance (ERI) model for school and student settings. The ERI scores on a 5 point Likert scale.

In total, N = 716 medical students completed the questionnaire (65.4% female, 34.6% male). 61% of the students were in their pre-clinical term, 39% in their clinical term.

The practicing medics sample had a total of N = 120 (60.2% female, 39.8% male).

## Results

The negative consequences of the ERI develop from domination of the “effort” in relation to the “reward” (ER-ratio > 1). The medical student sample had a validity total of N=680, with a minimum of 0.2 and a maximum of 2.2. The average throughout the sample was 0.91 with a standard deviation of 0.316. 66.9% of the sample was below the ERI-Cut off and 33.1 % were above the Cut off. The pre-clinical students did have an average of 0.935. The clinical students scored had an average of 0.884. The practicing medics sample had a validity total of N=1 06, with a minimum of 0.5 and a maximum of 2.5. The average throughout the sample was 1.36 with a standard deviation of 0.376. The major group of the sample was above the ERI-Cut off (90.1 %); just 9.9% of the sample were below.

## Conclusions

The average shows a large imbalance between effort and reward, with a clear domination of “effort” within the practicing medics’ sample. The medical student sample is below the ERI-Ratio Cut off (>1). The comparison between the averages of the pre-clinical students (0,935), the clinical students (0.884) and the practicing medics (1.36) show interesting dynamics. The lowest average (clinical section of the study) is located in a time when the students are getting more and more into social exchange by being split into different courses with practical tasks and exercises. In comparison, the highest average (practicing medics) is located in a time when social interaction with peers is reduced because workplace issues require more time in everyday work life.

## Funding

No funding was received for this project.

